# P-1737. Temporal Trends in Candidemia Mortality: Analysis of Contributing Factors in Costa Rica (2007-2023)

**DOI:** 10.1093/ofid/ofaf695.1908

**Published:** 2026-01-11

**Authors:** Juan Villalobos Vindas, Jose A Castro Cordero

**Affiliations:** Caja Costarricense de Seguro Social, San José, San Jose, Costa Rica; Caja Costarricense de Seguro Social, San José, San Jose, Costa Rica

## Abstract

**Background:**

Mortality trends in candidemia may reflect changes in epidemiology, diagnostic practices, and patient characteristics. We analyzed mortality rates and contributing factors in candidemia cases over a 17-year period in Costa Rica.

**Methods:**

We analyzed 2,128 candidemia cases from two tertiary hospitals (2007-2023), examining mortality rates at 7 and 30 days. We assessed temporal trends, the impact of the COVID-19 pandemic, and associations between mortality and various clinical factors including growth velocity, hospital service, and patient demographics.

**Results:**

Overall mortality was 22.8% at 7 days and 42.1% at 30 days. No statistically significant temporal trend was observed over the study period (regression analysis: p >0.05). However, mortality increased during the pandemic period (2020-2021) compared to pre-pandemic years (28.5% vs 21.8% at 7 days; 48.4% vs 40.9% at 30 days), with C. tropicalis showing the most dramatic increase (46.4% vs 26.9% at 7 days). Mortality varied significantly by hospital service (p< 0.001): highest in Emergency departments (38.9% at 7 days, 54.4% at 30 days) and lowest in Surgical services (14.3% at 7 days, 33.6% at 30 days). Growth velocity showed a strong inverse correlation with survival: isolates growing in < 24 hours had significantly higher mortality (29.9% at 7 days, 50.0% at 30 days) than those growing in >72 hours (18.3% at 7 days, 42.6% at 30 days). Age was a critical determinant of outcome, with mortality exceeding 70% at 30 days in patients ≥80 years. Candidemia involving multiple species (mixed candidemia) showed slightly lower mortality (19.2% vs 22.9% at 7 days) than monomicrobial infections.

**Conclusion:**

While candidemia mortality in Costa Rica remained stable over the study period, significant increases occurred during the COVID-19 pandemic. The strong associations between growth velocity and mortality, as well as variations by hospital service, provide novel insights for risk stratification. These findings can guide the development of targeted prevention strategies and treatment protocols, particularly for high-risk settings such as Emergency departments and for vulnerable populations such as the elderly.

**Disclosures:**

All Authors: No reported disclosures

